# Gene delivery in mosquitos with a vesicular stomatitis virus vector

**DOI:** 10.1016/j.isci.2025.113510

**Published:** 2025-09-07

**Authors:** Yuhang Zhang, Xueli Wang, Huiying Qi, Fei Yuan, Hongyue Li, Qiang Hu, Zhen Zou, Aihua Zheng

**Affiliations:** 1 State Key Laboratory of Animal Biodiversity Conservation and Integrated Pest Management, Institute of Zoology, Chinese Academy of Sciences, Beijing 100101, China; 2 College of Life Sciences, University of Chinese Academy of Sciences, Beijing 101408, China; 3 College of Life Science, Henan Normal University, Xinxiang 453007, China; 4 College of Life Science, Hebei University, Baoding 071002, China

**Keywords:** Biological sciences, Neuroscience, Virology, Genetic engineering

## Abstract

Gain- and loss-of-function techniques are essential for comprehensive investigation of gene function. In mosquitoes, effective loss-of-function (LOF) methods such as RNA interference and gene knockout are available. However, convenient and practical methodologies for gain-of-function (GOF) are currently lacking. Here, we developed the vesicular stomatitis virus (VSV) vector as a GOF delivery system, which efficiently delivers target genes in mosquito *Aedes aegypti* without affect their fitness. Importantly, by tactically altering the insertion site of target genes within the VSV vector, we can control the relative levels of transcription. This VSV-mediated method resulted in the corresponding phenotypic changes in mosquitoes overexpressing target genes compared to controls. Additionally, we discovered that VSV could infect various orders of insects, including fruit flies (Diptera), fall armyworms (Lepidoptera), and beetles (Coleoptera), highlighting its potential as a versatile gene delivery platform across insect species.

## Highlights

• VSV infects mosquitoes via multi-routes without impairing ontogenetic development
• VSV serves as an efficient and versatile platform for GOF studies in mosquitoes
• Target gene expression levels can be modulated by VSV insertion sites
• The VSV platform enables exogenous gene expression in multiple insect orders

## Graphical abstract

**Figure:**
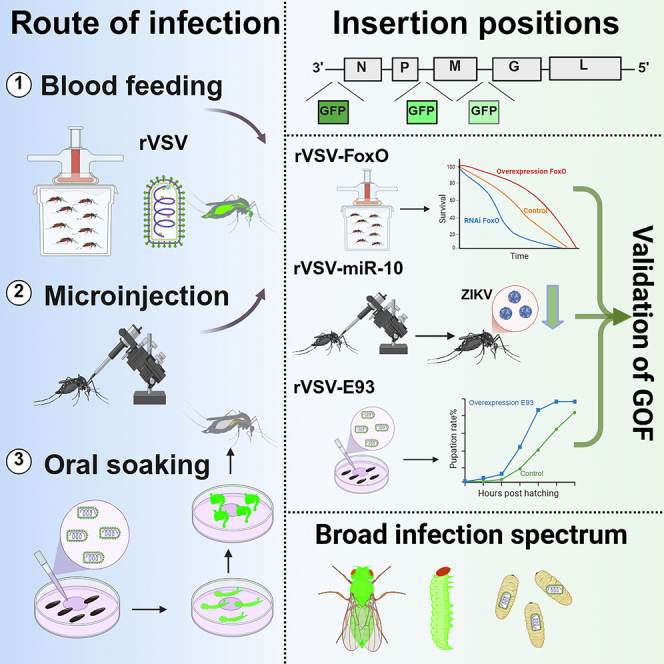

## Introduction

Female mosquitoes transmit numerous pathogens of human diseases due to their cyclic blood feeding, which is required for ovarian development and egg maturation. The mosquito *Aedes aegypti* is the competent vector for dengue, yellow fever, and Zika, posing a serious threat to human health.^1^^,^^2^^,^^3^ Therefore, understanding the functional roles of genes associated with mosquito physiology and vector competence is essential for revealing the underlying mechanisms of pathogen acquisition and transmission by mosquito vectors. This knowledge is also vital for developing novel strategies to control mosquito populations and eventually combating devastating human diseases.

Studies of gene function would greatly benefit from the utilization of genetic techniques that are informed by mosquito genome. Current methods for investigating gene function in mosquitoes include gain-of-function (GOF) and loss-of-function (LOF) approaches. RNA interference (RNAi) or clustered regularly interspaced short palindromic repeat (CRISPR)-Cas9-mediated site-directed mutagenesis is considered as the commonly used methods to explore LOF phenotypes by knocking down or out the transcripts of the target genes within the mosquitoes, respectively. For example, RNAi is widely used to study the role of genes in development, metabolism, and immune during the reproductive cycles of *Ae. aegypti*.^4^^,^^5^^,^^6^^,^^7^^,^^8^ CRISPR-Cas9 has enabled the construction of gene knockout strains to investigate key gene regulatory pathways in mosquitoes, such as sex determination, olfaction, behavior, and metamorphosis.^9^^,^^10^^,^^11^^,^^12^ For GOF studies, CRISPR-Cas9-mediated site-specific recombination or transactivation and binary expression systems (such as the UAS/Gal4 and QUAS/QF2) are two well-known systems to express exogenous genes within mosquitoes.^13^^,^^14^^,^^15^^,^^16^^,^^17^ However, the availability of these two methods is still limited for many researchers working with *Ae. aegypti* due to the demands of the high levels of technical expertise. Furthermore, construction of stable gene-edited mosquito strains for each gene of interest is not only labor-intensive but also time-consuming.^18^ This process, from egg injection to establishing stable homozygous strains, can span up to 3 months.^12^ Additionally, manipulating the expression levels of certain genes, particularly those critical to specific development stages, can be lethal, rendering the establishment of stable mosquito strains impossible.^19^^,^^20^ Thus, the convenient and practical techniques for GOF studies in mosquitoes are urgently needed.

Viral vector transduction system emerges as a promising method for GOF research, as it offers several advantages, including convenient handling, high efficiency in transduction, and sustained gene expression.^21^^,^^22^ The mosquito densoviruses (MDVs) and baculovirus expression systems are the well-studied viral vectors utilized for gene overexpression in mosquitoes. MDVs are non-enveloped, small DNA viruses, which are naturally transmitted and maintained within mosquito populations, infecting a wide range of mosquito organs and tissues. MDVs have been extensively studied as overexpression vectors in mosquitoes. They not only facilitate RNA overexpression through injection in adults but also enable the delivery of small RNAs to larvae via oral ingestion.^23^^,^^24^ However, MDVs are limited in their capacity to package exogenous sequences no more than 400 bp in length, which significantly restricts their utility as delivery vectors.^25^ In contrast, baculoviruses such as *Autographa californica* multiple nucleopolyhedrovirus, which possess large circular double-stranded DNA genomes, offer substantial transgenic capacity, potentially accommodating over 100 kb of foreign DNA.^21^^,^^26^ The virus carrying the green fluorescent protein (*GFP*) reporter gene is able to infect mosquitoes at various developmental stages.^21^ Unfortunately, baculovirus acts as a lethal pathogen to mosquito larvae, potentially leading to covert infection and reducing the reproductive capacity of infected female mosquitoes.^27^^,^^28^^,^^29^^,^^30^ The adverse effects of baculovirus on mosquito growth and development limit its applicability for gene function studies. Hence, it is required to develop the additional virus-mediated gene delivery systems that allow the insertion of exogenous sequences with different lengths and have no impact on the fitness of mosquitoes.

Vesicular stomatitis virus (VSV), a single-stranded negative-sense RNA virus, is a prototype virus of the *Rhabdoviridae* family. It has a relatively small genome (11 kb) encoding only five structural proteins, which facilitates genetic manipulation. VSV exhibits a broad infection spectrum, capable of infecting mammals as well as arthropods, such as mosquitoes, sandflies, flies, and midges.^31^^,^^32^^,^^33^^,^^34^ Since the infectious VSV was successfully recovered from cDNA using reverse genetics technology, it has been widely utilized as a vector in vaccine development and gene therapy.^35^^,^^36^^,^^37^^,^^38^^,^^39^ In this study, we developed the VSV-based gene delivery systems to investigate the GOF of genes in *Ae. aegypti*. To assess the effectiveness of this system, we focus on the overexpression of two heterologous genes (*forkhead box protein O* [*FoxO*] and *ecdysone-induced protein 93* [*E93*]) and one non-coding RNA (miR-10) for functional analysis in mosquitoes. In addition, we confirmed that the VSV vector was capable of infecting other insects, suggesting a broad spectrum of susceptibility. Our results underscore that VSV could act as a promising tool for exploring gene function in a variety of insect species.

## Results

### Adult *Ae. aegypti* mosquitoes were susceptible to recombinant VSV

**Figure 1:**
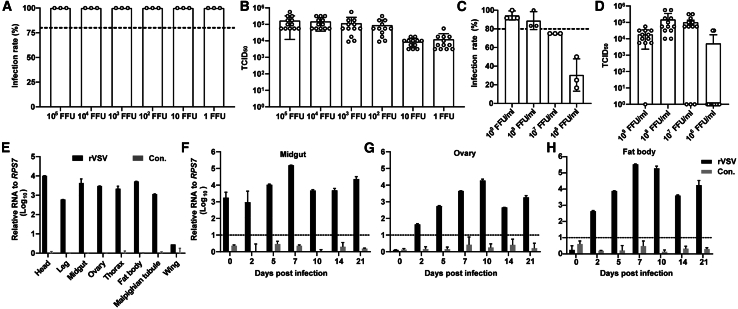
Susceptibility of adult *Ae. aegypti* mosquitoes to rVSV (A–D) Twelve mosquitoes were microinjected (A and B) or blood-fed (C and D) with increasing doses of rVSV (A and C) Infection rate was determined as the number of infected mosquitoes relative to the total number of mosquitoes (*n* = 3). (B and D) The viral titers of individual mosquito were determined by TCID_50_ assay at 7 dpi. Data are presented as mean ± SD. (E–H) Tissue preference of rVSV infection in mosquitoes. Six-day-old female adults were blood-fed with 1 × 10^8^ FFU/mL rVSV. Total RNAs from eight tissues of fifteen mosquitoes were extracted at 7 dpi, and the viral RNAs were detected by real-time qPCR (E). Total RNAs from midguts (F), ovaries (G), and fat bodies (H) of eight mosquitoes were extracted at different days after infection, and the viral RNA was detected by real-time qPCR. Control (Con.) represents uninfected mosquito groups.

To investigate the susceptibility of mosquitoes to VSV, we constructed an antigenomic VSV plasmid containing the *GFP* coding sequence upstream of the nucleoprotein (N) gene. To enhance vector safety, the M51 site in the matrix (M) gene was deleted to reduce VSV toxicity.^40^^,^^41^ The resulting recombinant VSV (rVSV) was subsequently recovered and utilized to infect lab-adapted *Ae. aegypti* mosquitoes on the 6th day post eclosion, either through microinjection or blood feeding. Microinjection achieved a 100% infection rate, irrespective of the rVSV dose (Figure 1A). To determine viral titers in infected mosquitoes, supernatants from mosquito homogenates were used to infect Vero cells on the 7th day post infection. The average viral titers of individual mosquitoes exceeded 10^4^ TCID_50_ (50% tissue culture infectious dose) following microinjection with an rVSV dose as low as one focus-forming units (FFU) (Figure 1B). Compared to microinjection, blood feeding represents a simpler and safer infection method. However, the infection rate gradually decreased as the virus dose dropped. At a dose of 10^7^ FFU per milliliter (FFU/mL), the infection rate fell below 80%. Thus, 10^8^ FFU/mL was identified as the optimal dose for blood feeding (Figure 1C). Importantly, there was no significant difference in viral loads in individual mosquitoes regardless of the infection route (Figure 1D). These findings demonstrate that adequate doses of rVSV can achieve robust infection rates in *Ae. aegypti* mosquitoes through both microinjection and blood-feeding methods.

To further elucidate the tissue preference of rVSV infection, *Ae. aegypti* mosquitoes were dissected 7 days after infection following blood meal. Quantitative real-time polymerase chain reaction (real-time qPCR) and TCID_50_ assays were performed to assess viral loads in eight distinct body parts: head, leg, midgut, ovary, thorax, fat body, Malpighian tubule, and wing (Figures 1E and S1). We found that the infection route—blood feeding or microinjection—did not significantly affect rVSV tissue tropism or viral titers in *Ae*. *aegypti* mosquitoes at 7 days post infection (dpi). High RNA levels of rVSV were detected in all examined tissues except the wings, with the highest viral loads observed in the head (Figure 1E). GFP signals, indicative of rVSV infection, were further confirmed via confocal imaging in the brain, leg, and fat body (Figure S2). We then focused on the midgut, fat body, and ovaries—key tissues involved in mosquito metabolism, immunity, and reproduction—to examine the persistence of rVSV infection. Real-time qPCR analysis revealed that rVSV RNA levels peaked at day 7 in the midgut and fat body and day 10 in the ovary. Notably, rVSV RNA remained consistently detectable in these tissues up to 21 dpi (Figures 1F–1H).

To assess the potential for vertical transmission of rVSV within *Ae. aegypti*, we collected eggs laid by infected mosquitoes via both microinjection or blood-fed routes, as well as the larvae and adults that hatched from those eggs. Real-time qPCR and TCID_50_ assays revealed no detectable viral RNAs or infectious viruses in any of the tested mosquito samples (Figure S3). These findings suggest that although rVSV is able to effectively infect ovarian tissue, it is not vertically transmitted to offspring, thus ensuring the biosafety of rVSV as a delivery vector. Overall, these results highlight rVSV as a safe, efficient, and non-tissue-restricted delivery platform suitable for a variety of applications.

### rVSV infection has no impact on the fitness of *Ae. aegypti*

**Figure 2:**
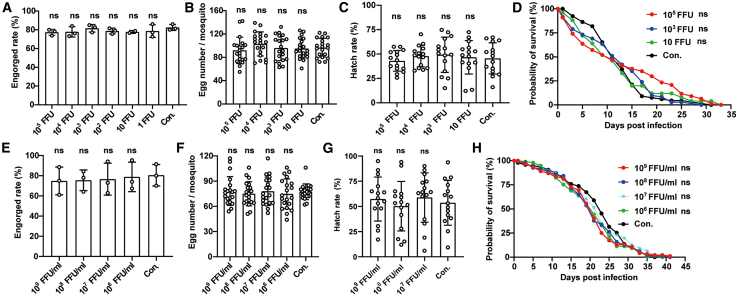
Effect of rVSV infection on reproduction and survival of *Ae. aegypti* Mosquitoes were infected with indicated doses of rVSV via microinjection (A–D) or blood feeding (E–H). (A and E) Engorgement rate of 50 adults at 7 dpi (chi-square test followed by Bonferroni test). (B and F) The number of eggs laid per mosquito (*n* = 20, Kruskal-Wallis test followed by Dunn’s post hoc test). (C and G) The hatching rates of mosquito eggs (*n* = 15, chi-square test followed by Bonferroni test). (D and H) The survival curves of mosquitoes (*n* = 50, Kaplan-Meier curves and log rank test). Con. represents mosquito groups microinjected or blood-fed with medium. Data are presented as mean ± SD. ns, not significant. These results are representative of three independent experiments.

To assess the impact of rVSV infection on the reproduction and survival of *Ae. aegypti* mosquitoes, we monitored blood engorgement rates, egg production, hatching rates, and lifespan. Seven days after microinjection with rVSV, mosquitoes were blood-fed to evaluate their engorgement rates, which showed no significant difference compared to the uninfected group (Figure 2A). Additionally, egg production and hatching rates remained unchanged compared to those of the control group after microinjection (Figures 2B and 2C). Survival assays revealed that there was no impact on lifespan for mosquitoes microinjected with 10^5^ FFU, 10^3^ FFU, and 10 FFU of rVSV (Figure 2D). Similarly, infection of mosquitoes with rVSV through blood feeding did not alter engorgement rates, survival rates, egg numbers, or hatching rates (Figures 2E–2H). These findings suggest that rVSV does not affect the fitness of infected mosquitoes compared to uninfected controls, highlighting its potential as a vector for gene expression in mosquitoes.

To achieve a high infection rate while minimizing the impact on mosquito survival, we selected a dose of 10^3^ FFU per mosquito for microinjection and 10^8^ FFU/mL for blood feeding in subsequent studies.

### The expression levels of the target gene vary with the change of insertion positions

**Figure 3:**
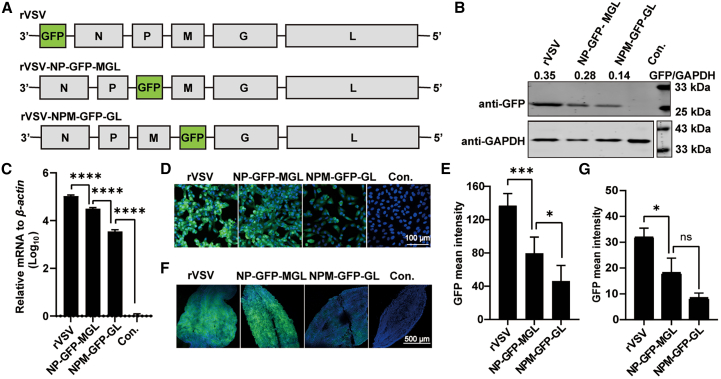
The insertion positions of *GFP* gene affected its expression level (A) Schematic diagram of *GFP* gene insertion positions. (B) Vero cells were infected with rVSVs at an MOI of 0.1. Western blotting of the cell lysates was performed at 24 h after infection using anti-GFP or anti-GAPDH mouse antibodies. Band quantification was carried out using Image Studio software (Licor), and the numbers indicated the ratio of GFP to GAPDH. (C) Real-time qPCR of *GFP* expression levels relative to *β-actin* at 24 h after rVSV infection. Data are presented as mean ± SD of triplicate measurements. Con. represents mock-infected Vero cells. (D) GFP fluorescence in rVSV-infected Vero cells was detected at 24 h after infection under a fluorescence microscope. (F) Mosquitoes were blood-fed with indicated rVSVs, and their midguts were visualized under fluorescence microscopy at 5 dpi. GFP was shown in green, and the nucleus was stained with Hoechst 33342 (blue). (E and G) Quantitative results of the GFP fluorescence intensity in (D) or (F). Kruskal-Wallis test followed by Dunn’s post hoc tests. ∗*p* < 0.05, ∗∗∗*p* < 0.001, ∗∗∗∗*p* < 0.0001. ns, not significant. These results are representative of three independent experiments.

Previous studies have shown a gradient decrease in mRNA abundance as genes are positioned further from the 3′ promoter of VSV, following the order N > P > M > G > L.^42^^,^^43^^,^^44^ To monitor the expression levels of target gene, we generated two additional constructs where the *GFP* coding sequence was inserted between P and M and between M and G, respectively. These resulting viruses, named rVSV-NP-GFP-MGL and rVSV-NPM-GFP-GL, along with rVSV, were used to infect Vero cells at a multiplicity of infection (MOI) of 0.1, and GFP expression was assessed 24 h post infection (Figure 3A). Quantitative experiments and western blot analysis demonstrated a gradual decrease in GFP expression at both mRNA and protein levels (Figures 3B and 3C). GFP protein levels in rVSV-infected cells were approximately 2.5-fold higher than those in rVSV-NPM-GFP-GL-infected cells. Additionally, as the *GFP* gene was positioned further from the 3′ end, the average fluorescence intensity decreased accordingly (Figures 3D and 3E). To evaluate GFP expression *in vivo*, mosquitoes were blood-fed with the three viruses, and midguts were visualized under a confocal microscope at 6 dpi. GFP fluorescence in midguts infected with rVSV and rVSV-NP-GFP-MGL was significantly stronger than in those infected with rVSV-NPM-GFP-GL (Figures 3F and 3G). These findings highlight the versatility of the VSV vector in modulating target protein expressions in *Ae. aegypti* mosquitoes by strategically adjusting the insertion position of the target genes.

### Overexpression of FoxO in adult mosquitoes did not affect lifespan

**Figure 4:**
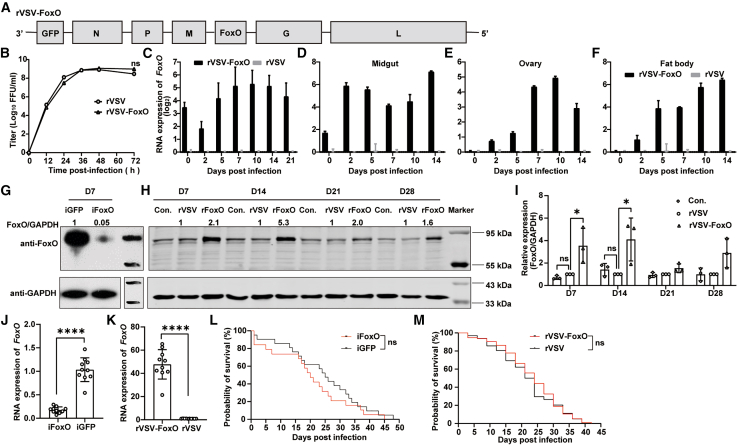
Overexpression of *FoxO* gene in adult mosquitoes did not affect lifespan (A) Schematic diagram of *FoxO* gene insertion position in the rVSV. (B) The growth curves of rVSV and rVSV-FoxO in Vero cells at an MOI of 0.01 (*n* = 3, unpaired t test, two-sided). (C–F) Six-day-old females were blood-fed with rVSV-FoxO or rVSV diluted to 1 × 10^8^ FFU/mL. Con. was fed with medium. Total RNAs were extracted from 8 mosquitoes (C), 8 midguts (D), 8 ovaries (E), or 8 fat bodies (F) at indicated dpi, and the *FoxO* expression was measured by real-time qPCR. Data are presented as mean ± SD of triplicate measurements (*n* = 3). (G and H) Ten mosquitoes per group were treated with iFoxO and iGFP via microinjection or infected with rVSV-FoxO (rFoxO) and rVSV via blood feeding. Western blotting of these mosquito homogenates was performed using anti-FoxO or anti-GAPDH antibodies. (I) Band intensities of FoxO in (H) were quantified using LiCor Image Studio and normalized to GAPDH. Results are shown as mean ± SD from three independent experiments (*n* = 3 biologically independent mixtures; two-way ANOVA test followed by Tukey’s post hoc tests). (J and K) Relative *FoxO* mRNA levels of ten mosquitoes at 7 days post treatment (unpaired t test, two-sided). (L and M) Survival curves of mosquitoes per group upon different treatments (Kaplan-Meier curves and log rank test). Error bars in graphs represent the mean ± SD. ∗*p* < 0.05, ∗∗∗∗*p* < 0.0001. ns, not significant; These results are representative of three independent experiments.

FoxO is known to intricately interact with the insulin/insulin-like growth factor signaling pathway, contributing to lifespan extension in fruit flies by enhancing antioxidant capacity, regulating energy metabolism, and improving adaptability.^45^^,^^46^ To elucidate FoxO’s role in mosquitoes, we inserted the *FoxO* gene between M and G, yielding the rVSV-FoxO virus (Figure 4A). Comparable growth curves between rVSV-FoxO and rVSV were observed in Vero cells, with peak titers of 6.2 × 10^8^ FFU/mL for rVSV-FoxO and 5.6 × 10^8^ FFU/mL for rVSV, suggesting that the exogenous gene did not affect virus amplification (Figure 4B). *Ae. aegypti* mosquitoes were then subjected to infection via blood feeding, and the mRNA levels of *FoxO* were monitored at 2, 5, 7, 10, 14, and 21 dpi. Quantitative analysis revealed a steady increase in *FoxO* RNA levels starting from day 5, reaching peak expression on day 10, and remaining at high levels until day 21 (Figure 4C). The *FoxO* transcript was further examined in various tissues at different post-infection time points (Figures 4D–4F). Robust expression of *FoxO* was detected in the midgut as early as 2 days after blood feeding (Figure 4D). Elevated levels of *FoxO* in the fat body and ovary persisted until days 5 and 7, respectively, possibly due to the midgut barrier in mosquitoes (Figures 4E and 4F). Additionally, the mRNA abundance of *FoxO* in all three tissues continued to be sustained until day 14 post infection.

To unveil the role of FoxO in mosquito longevity, we employed RNAi to knock down *FoxO* expression (iFoxO) or overexpressed *FoxO* via rVSV-FoxO infection. Western blot analysis showed a 95% reduction in endogenous FoxO protein levels with iFoxO treatment (Figure 4G). In contrast, mosquitoes blood-fed with rVSV-FoxO exhibited a significant increase in FoxO protein levels, peaking at day 14 post infection and surpassing controls by 5-fold (Figures 4H and 4I). Corresponding changes in FoxO mRNA levels were observed following both treatments (Figures 4J and 4K). Survival analysis revealed that iFoxO mosquitoes showed reduced survival rate before day 15 and between days 18 and 36, whereas FoxO overexpression improved survival during days 9–27 (Figures 4L and 4M). Notably, despite these temporal effects on mortality rates, neither FoxO knockdown nor overexpression significantly affected overall lifespan. These findings suggest that while FoxO expression influences survival rates during specific periods, it does not ultimately extend the overall lifespan of mosquitoes under our experimental condition, indicating a more intricate regulatory mechanism compared to fruit flies.

### Overexpression of *E93* gene by rVSV triggers precocious metamorphosis in mosquitoes

**Figure 5:**
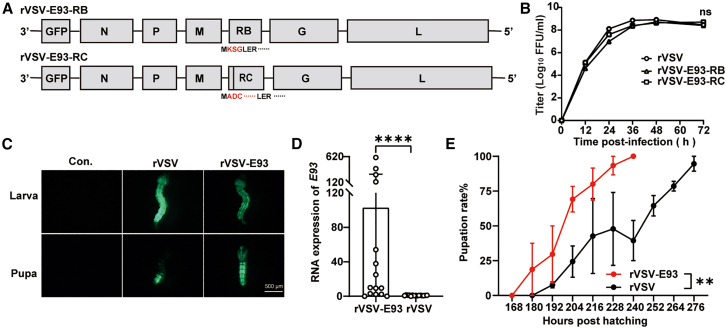
E93 overexpression shortens pupation time of larvae (A) Schematic diagram of the rVSV carrying *E93* gene. (B) The growth curves of rVSV-E93-RB, rVSV-E93-RC, and rVSV after infection of Vero cells at an MOI of 0.01 (*n* = 3, one-way ANOVA test). (C) Florescence images of larvae and pupa infected with rVSV and rVSV-E93. (D) Real-time qPCR analysis of *E93* transcripts in larvae infected with 10^9^ FFU/mL rVSV-E93 or rVSV (*n* = 15, unpaired t test, two-sided). Error bars in graphs represent the mean ± SD. (E) Pupation time of the larvae infected with rVSV-E93 or rVSV (unpaired t test, two-sided). Data are presented as mean ± SD of triplicate measurements. ns, not significant; ∗∗*p* < 0.01; ∗∗∗∗*p* < 0.0001. These results are representative of three independent experiments.

The E93 protein has been established as a critical determinant in metamorphosis of fruit flies, showing notably high expressional levels during the prepupa and pupa stages.^47^ Due to the alternative splicing of sequence in the 3′ non-coding region of the *E93* gene, it is categorized into two isoforms (*E93-RB* and *E93-RC*). We generated two rVSVs carrying these isoforms, designated as rVSV-E93-RB and rVSV-E93-RC, respectively (Figure 5A). Growth curve analysis revealed that both rVSV-E93-RB and rVSV-E93-RC viruses exhibited similar amplification trends to that of rVSV, reaching their highest titers approximately 48 h post infection, with no significant differences observed (Figure 5B). To investigate the infectivity of rVSV in mosquito larvae and the role of E93 in mosquito metamorphosis, rVSV-E93-RB and rVSV-E93-RC were mixed in a 1:1 ratio, and the resulting rVSV-E93 was used to infect mosquito larvae. We demonstrated successful infection of larvae through detecting GFP expression after oral soaking in a 10^9^ FFU/mL viral solution (Figure 5C). Consistently, the mRNA levels of *E93* in mosquito larvae were significantly increased following infection with rVSV-E93, as compared to infection with rVSV (Figure 5D). Continuous monitoring showed that *Ae. aegypti* larvae infected with rVSV-E93 underwent shorter pupation periods, with the majority completing pupation within 240 h post hatching. In contrast, pupation rates in the control group were consistently lower, with approximately 50% of larvae pupating between 240 and 276 h post hatching (Figure 5E). These results indicate that overexpression of E93 during the larval stage significantly reduces pupation duration and induces the precocious onset of metamorphosis. Overall, these findings highlight the infectivity of rVSV in *Ae. aegypti* larvae and its utility in expressing exogenous genes.

### Upregulation of miR-10 by rVSV reduced the susceptibility of adult mosquitoes to Zika virus

**Figure 6:**
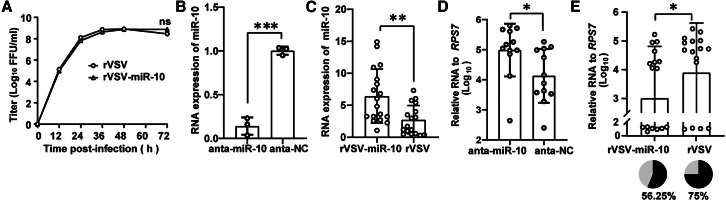
Delivery of miR-10 by rVSV reduced the susceptibility of adult mosquitoes to ZIKV (A) The growth curves of rVSV-miR-10 and rVSV after infection of Vero cells at an MOI of 0.01 (*n* = 3). (B and C) Mosquitoes were microinjected with anta-miR-10, antagomir-negative control (anta-NC), or rVSV-miR-10, rVSV within 24 h post eclosion. The RNA levels of miR-10 were examined by real-time qPCR. (D and E) Mosquitoes were blood-fed with ZIKV after microinjection with antagomirs or rVSVs. ZIKV RNA levels were measured using real-time qPCR at 7 dpi (*n* ≥ 12). Percentage represents the mosquito infection rate. Data are shown as mean ± SD. The figure illustrated one of three representative independent experiments. The experiments were repeated three times with similar results. ns, not significant; ∗*p* < 0.05; ∗∗*p* < 0.01; ∗∗∗*p* < 0.001 (unpaired t test, two-sided). These results are representative of three independent experiments.

MicroRNAs (miRNAs) are endogenous non-coding small RNAs that play a crucial role in post-transcriptional regulation. They function by specifically targeting host RNAs, thereby modulating the expression of cell factors that either inhibit or facilitate viral infections.^48^ Previous studies have demonstrated that miRNAs exhibited differential expression in response to Zika virus (ZIKV) infection, with miR-10 being significantly upregulated at 6 dpi.^49^ To further confirm the function of rVSV in the delivery of miRNAs, we select miR-10 as the target in this investigation. We engineered the rVSV vector to incorporate miR-10 sequence between M and G and successfully recovered the rVSV-miR-10 virus with a titer of 7.5 × 10^8^ FFU/mL (Figure 6A). To evaluate the impact of miR-10 levels on ZIKV infection, mosquitoes were microinjected with either miR-10 antagomir (anta-miR-10) or 10^3^ FFU of rVSV-miR-10 within 24 h post eclosion. The results showed that anta-miR-10 significantly reduced miR-10 expression, whereas rVSV-miR-10 facilitated miR-10 overexpression in *Ae. aegypti* (Figures 6B and 6C). The mosquitoes were subsequently subjected to infection with ZIKV (10^4^ FFU/mL) via blood feeding, and ZIKV infection levels were assessed at 7 dpi. The increased expression of miR-10 resulted in a decrease in viral RNA levels of approximately one log, whereas the reduction of miR-10 had the opposite effect. Mosquitoes administered with rVSV-miR-10 exhibited a ZIKV infection rate of 56.25%, contrasting with the 75% infection rate observed in the control group (Figures 6D and 6E). This difference suggests that miR-10 overexpression effectively attenuates the susceptibility of *Aedes* mosquitoes to ZIKV. These findings underscore the potential of rVSV for delivering miRNAs into mosquitoes, offering new insights for developing novel antiviral strategies against mosquito-borne diseases.

### The broad spectrum of rVSV vector delivery in insects

**Figure 7:**
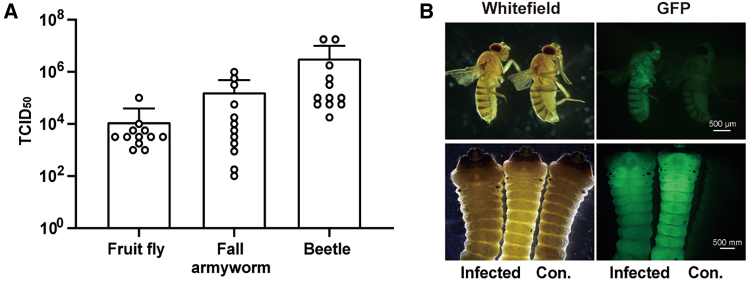
The broad-spectrum susceptibility of rVSV in insects (A) The rVSV was microinjected into fruit flies (5 × 10^4^ FFU), 3-day-old fall armyworm larvae (1.5 × 10^5^ FFU), and beetle larvae (5 × 10^7^ FFU). The viral titers of individual sample were determined by TCID_50_ assay at 7 dpi (*n* = 12). Data are shown as the mean ± SD. (B) Visualization of GFP fluorescence in rVSV-infected adult fruit flies (up) and beetle larvae (down). The infected insects (infected) were in the left, and untreated control (Con.) was in the right. These results are representative of three independent experiments.

Previous researches have demonstrated the broad infectivity spectrum of rVSV, which includes insects such as sand flies, black flies, and midges.^50^^,^^51^^,^^52^ To verify whether rVSV could act as an effective gene delivery system in other species beyond mosquitoes, we evaluated its infectivity in three additional insect species: fruit flies (Diptera), fall armyworm larvae (Lepidoptera), and beetle larvae (Coleoptera). Our findings revealed that all the tested insects could be effectively infected with rVSV through microinjection. Seven days after infection, viral titers in individual sample, as determined by TCID_50_ assay, ranged from 1.1 × 10^4^ to 3.2 × 10^6^ TCID_50_/mL (Figure 7A). The robust infection was further evidenced by widespread GFP fluorescence observed throughout the bodies of fruit fly and beetle larvae under fluorescence microscopy (Figure 7B). These findings ascertain the versatility of rVSV as a delivery vector in various insect species.

## Discussion

In this study, we introduced a rhabdovirus-based vector that enables effective and safe overexpression of target genes in *Ae*. *aegypti* mosquitoes with minimal safety concerns. Using this vector, we investigated the roles of overexpressed genes such as *E93*, *FoxO*, and the non-coding RNA miR-10 in relation to *Ae. aegypti* development, survival, and susceptibility to viruses. As expected, the overexpression of *E93* led to the premature onset of metamorphosis consistent with the phenotypes observed in *Tribolium castaneum* upon *E93* upregulation and contrary to the effects of *E93* knockdown via RNAi.^53^ Moreover, the overexpression of miR-10 resulted in phenotypes opposite to those observed in the LOF studies. Notably, overexpression of FoxO in mosquitoes did not produce the expected phenotype of lifespan extension, as observed in *Drosophila*.^54^^,^^55^ Similarly, RNAi-mediated downregulation of this gene also had no significant effect on lifespan. These findings suggest that mosquito longevity is regulated by more complex factors. In addition, the expression levels of target genes could be quantitatively regulated by strategic manipulation of their insertion sites on the vector. Notably, our system not only demonstrated the efficacy in delivering genes in *Ae. aegypti* but also exhibited promising application prospects across various insect species.

While the CRISPR-Cas system has enabled GOF studies of target genes by generating mosquito strains with gene knockin or transactivation, its practical application remains constrained by the time-consuming and labor-intensive process required to construct these specific mosquito strains.^56^^,^^57^ In contrast, the process from recovery to amplification of rVSV can be rapidly completed, typically within 2–3 weeks. Overexpression of genes delivered by rVSV can be detected as early as 2 days post viral infection, with protein levels persisting for up to 28 days. Moreover, since VSV can infect both adult mosquitoes and larvae, it offers advantages for research into gene functions across different stages of the mosquito life cycle without impacting growth and development.

In mosquito research, several virus vector systems such as baculovirus and MDVs have already been employed for delivering target genes.^21^^,^^23^^,^^26^ However, baculovirus can induce covert infections in mosquitoes, potentially compromising their growth and development.^30^ MDVs exhibit species specificity in infecting mosquitoes and are limited in the size of genes they can deliver, thereby restricting its application.^25^ The VSV-based delivery system can overcome these limitations, providing safe, efficient, and stable overexpression of target genes with fewer size constraints. Our study demonstrated that rVSV did not affect the reproduction and survival of the infected mosquitoes and did not vertically transmit to subsequent generations through eggs, ensuring safety for mosquitoes and the environment. The advantage of VSV over MDVs is its large cargo capacity. For instance, rVSV-E93 accommodated the entire 4.6 kb open reading frame (ORF) of the *E93* gene while maintaining titers comparable to the original VSV. Additionally, the VSV vector can carry multiple ORFs at five different positions in its genome, enabling simultaneous expression of multiple genes and enhancing its versatility in insect applications. In future studies, we can try to integrate short hairpin RNA sequences into the VSV vector to enable LOF studies; however, the availability of these approaches needed to be confirmed further.

Based on our experimental findings, rVSVs could effectively infect adult mosquitoes via both microinjection and blood-feeding routes. Following microinjection with rVSV, mosquitoes exhibited a 100% infection rate, even when administered the minimal dose of 1 FFU. For blood-feeding method, a higher dose is required to achieve infection rates exceeding 80%, with virus titers in infected mosquitoes reaching up to 10^5^ TCID_50_. Due to its ease of operation and harmlessness to mosquitoes, blood-feeding method is considered as a more convenient approach for VSV infection. In mosquitoes, the availability of genetic-based methods for exploring gene function in larval stage is largely limited due to the challenges associated with larvae fixation and the detrimental effects on the survival of larvae when removed from water. VSV, however, could successfully infect larvae through oral soaking, providing a powerful tool for studying the genetic functions of mosquito larvae. Except for mosquitoes, VSV was also able to infect diverse insect species including fruit flies, fall armyworm larvae, and beetle larvae through microinjection, indicating its broad applicability across multiple insect species as a versatile gene delivery tool.

In summary, our study introduces a safe, efficient, stable, and non-tissue-specific VSV vector, enhancing the gene function studies in *Ae. aegypti* mosquitoes. By manipulating the insertion sites of target genes, we can precisely control their expression levels. Importantly, the VSV vector facilitates gene expression not only in mosquitoes but also in other insect species, underscoring its potential as a promising tool for insect gene delivery.

### Limitations of the study

CRISPR knockin technology integrates exogenous genes into the mosquito genome to establish genetically modified strains, enabling stable and persistent expression of the transgene throughout the mosquito life cycle. Although exogenous proteins delivered via the VSV-based strategy are detectable throughout the adult lifespan, the VSV-mediated gene delivery system relies on viral replication efficiency to achieve transgene expression, resulting in variable protein expression levels across different developmental stages (as illustrated in Figures 4H and 4I). Additionally, VSV cannot achieve vertical transmission in mosquitoes (as illustrated in Figure S3). Consequently, in scenarios necessitating life cycle-spanning expression (from larval to adult stages) or heritable transmission across generations (e.g., mosquito population control), CRISPR knockin remains the superior strategy.

It should be noted that, as a newly developed viral vector delivery system, VSV exhibits several limitations in the study of mosquito’s gene functions. One major drawback is that VSV has to overcome a mosquito’s immune barrier in order to deliver the target gene, which results in a delay in target gene expression. Additionally, the target gene is continuously expressed without the possibility of termination once the virus infected the host. This leads to the inability to precisely regulate gene expression levels at specific developmental stages in mosquitoes. However, small-molecule viral inhibitors, notably ribavirin, an analog of guanosine nucleotide, and favipiravir, an RNA polymerase inhibitor, have exhibited encouraging results in inhibiting the replication of rhabdoviruses *in vitro*, offering a glimmer of hope in this regard.^58^^,^^59^ In the future, the ongoing development of small-molecule viral inhibitors may enable the temporal control of VSV-mediated target gene expression.

## Resource availability

### Lead contact

Requests for further information and resources should be directed to and will be fulfilled by the lead contact, Aihua Zheng (zhengaihua@ioz.ac.cn).

### Materials availability

All unique/stable reagents generated in this study are available from the lead contact with a completed materials transfer agreement.

### Data and code availability

• All original western blot images, confocal microscopy images, along with processed real-time qPCR analysis data used for this study, have been deposited at Mendeley Data: https://data.mendeley.com/datasets/3pb4vh7hjz/1.
• All plasmid sequences generated in this study are available in the GenBase: GB0006180 https://ngdc.cncb.ac.cn/genbase.
• Additional information reported in this paper is available via contacting the lead contact.

## Acknowledgments

This project was funded by Basic Frontier Scientific Research Program of the 10.13039/501100002367Chinese Academy of Sciences (ZDBS-LY-SM027-05), 10.13039/501100001809National Natural Science Foundation of China Major Program (32090024, 32370522, and 32370518), State Key Laboratory of Animal Biodiversity Conservation and Integrated Pest Management (grant no. SKLA2507), and the Initiative Scientific Research Program, Institute of Zoology, 10.13039/501100002367CAS (2024IOZ0105).

## Author contributions

A.Z. and Z.Z. conceived and supervised the project. Y.Z., X.W., Q.H., F.Y., H.L., and H.Q. performed the experiments and analyzed the data. A.Z., F.Y., X.W., and Y.Z. wrote the manuscript with the input of all authors.

## Declaration of interests

The authors declare no competing interests.

## STAR★Methods

### Key resources table

**Table:** 

REAGENT or RESOURCE	SOURCE	IDENTIFIER
**Antibodies**		

FoxO (Polyclonal)	GenScript	RRID: AB_3714716
GAPDH (Monoclonal)	Abbkine	Cat# ABL1020; RRID: AB_3714704
GFP antibody (Monoclonal)	Abbkine	Cat# ABT2020; RRID: AB_3714713

**Bacterial and virus strains**		

Zika virus (ZIKV MR766 strain)	GenBank	HQ234498
Vesicular stomatitis virus	Beijing SYKM Gene Biotechnology Co., Ltd	NC_038236.1
Stbl3	Beijing Genesand Biotech	SCC04

**Chemicals, peptides, and recombinant proteins**		

MiR10 antagomirs	GUANGZHOU RIBOBIO CO., LTD.	N/A
Trizol	Beijing Genesand Biotech	Cat. 15596018
Hoechst 33342	Solarbio Life Sciences	Cat. C0031
One-Step TB Green PrimerScript RT-PCR Kit	Takara	Cat. RR066A
Mir-X miRNA First-Strand Synthesis Kit	Takara	Cat. 638313
TB Green Premix Ex Taq II (Tli RNaseH Plus)	Takara	Cat. RR820A

**Deposited data**		

Raw data	Mendeley Data	http://doi.org/10.17632/3pb4vh7hjz.1
Plasmid sequences	GenBase	GB0006180

**Experimental models: Cell lines**		

Vero	Shenzhen Kangtai Biological Products Co., Ltd.	N/A
293T	ATCC	CRL-3216

**Experimental models: Organisms/strains**		

*Aedes aegypti*	UGAL/Rockefeller strain	N/A
Fruit flies		N/A
Fall armyworm		N/A
Longicorn		N/A

**Recombinant DNA**		

pVSV-FoxO	GUANGZHOU RIBOBIO CO., LTD.	C_AA111402.1
pVSV-E93 RB	GUANGZHOU RIBOBIO CO., LTD.	C_AA111400.1
pVSV-E93 RC	GUANGZHOU RIBOBIO CO., LTD.	C_AA111401.1
pVSV-miR10	GUANGZHOU RIBOBIO CO., LTD.	C_AA111404.1

### Experimental model and study participant details

#### Ethics statement

All viruses and insect experiments were conducted in accordance with bioethical principles under Biosafety Level 2 (BSL2) conditions and approved by the Animal Care and Use Committee of the Institute of Animal Research, Chinese Academy of Sciences (IOZ-IACUC-2022-255).

The VSV (GenBank: NC_038236.1) and ZIKV MR766 strain (GenBank: HQ234498) experiments were performed under biosafety BSL2.

#### Cells and antibodies

Vero cells (African green monkey kidney cells) and 293T cells (Human embryonic kidney cells) were maintained in Dulbecco’s modified Eagle’s medium (DMEM, HyClone) supplemented with 8% fetal bovine serum (FBS), 1% L-glutamine, and 1% penicillin-streptomycin at 37°C and 5% CO_2_. The FoxO antibody was purified by GenScript (China). Antibodies against GAPDH (Cat. ABL1020) and GFP (Cat. ABT2020) were purchased from Abbkine (China).

### Method details

#### Construction and rescue of rVSV viruses

The VSV vector plasmid (pVSV) was designed and constructed as previously described.^60^ It contains the VSV genome of Indiana strain, with a methionine deletion at position 51 in the *Matrix (M)* gene and the *green fluorescent protein* (*GFP*) coding sequence inserted upstream of the *Nucleoprotein (N) gene*. The coding sequences (CDS) for *FoxO* (GenBank: ABK76646.1), *E93* (GenBank: AAEL004572-RB and AAEL004572-RC) and pre-miR-10 (VectorBase: AAEL018774) were synthesized by Beijing SYKM Gene Biotechnology Co., Ltd or GUANGZHOU RIBOBIO CO., LTD. (China) and inserted between the M and G genes of the pVSV plasmid to generate the full-length VSV plasmids pVSV-FoxO, pVSV -E93-RB, pVSV -E93-RC, and pVSV -miR-10. Additionally, homologous recombination was employed to remove the GFP sequence upstream of N, followed by re-insertion of the GFP coding sequence either between P and M or between M and G. These constructs were designated as pVSV-NP-GFP-MGL and pVSV-NPM-GFP-GL, respectively.

Recombinant VSVs were generated using a reverse genetics approach. HEK293T cells cultured in 6 cm dishes were co-transfected with the full-length VSV plasmids (1.8 μg) described above, along with supporting plasmids encoding T7 polymerase (8.1 μg) and the VSV proteins N (1.28 μg), P (0.64 μg), M (0.17 μg), G (0.17 μg), and L (0.17 μg) proteins, using the calcium phosphate transfection method. Virus-containing supernatants were harvested upon observation of cytopathic effects and subsequently amplified in Vero cells.

#### Focus-forming assay and virus growth curve

A focus-forming assay was used to determine the viral titer of rVSVs in Vero cells. Vero cells were seeded at a density of 1.5x10^4^ cells per well in 96-well plates 24 hours prior to infection. The viruses were serially diluted in DMEM supplemented with 2% FBS at a 1:10 ratio and then incubated with the cells for 3 hours at 37°C. After incubation, the cells were washed once and then maintained at 28°C in fresh DMEM containing 2% FBS, 1% penicillin/streptomycin, and 20 mM NH_4_Cl. Twenty hours post-infection, GFP-positive cells were counted using a fluorescent microscope.

Virus growth curves were generated by infecting Vero cells in T75 flasks with rVSVs at a MOI of 0.01. Supernatants were collected every 12 hours post-infection, and viral titers were quantified using focus-forming assays in Vero cells.

#### Tissue culture infectious dose (TCID_50_) assay

The viral load in arthropods was measured using the TCID_50_ assay. Briefly, on the 7th day post-injection, the arthropods were homogenized in 1 ml of DMEM containing 2% FBS and the supernatant was collected after centrifugation. Then, the supernatant was serially diluted 10-fold and added to Vero cells in 96-well plates in octuplicate (100 μl per well). After incubation at 28°C for 3 days, fluorescence of infected cells was determined using the 4G2 antibody under a fluorescence microscope. The titers were expressed as log_10_ TCID_50_ and calculated using the Reed–Muench method.

#### Insect infection experiment

The *Ae. aegypti* (UGAL/Rockefeller strain) mosquitoes were cultured at 28°C with 70% humidity, as described previously.^61^^,^^62^ Larval mosquitoes were fed a diet composed of mice food, albumin, and yeast extract (1:1:1), while adult mosquitoes were provided with water and a 10% (wt/vol) sucrose solution. Six-day-old female mosquitoes were starved for 20 hours prior to oral infection. For the infection process, viruses from Vero cell supernatant were diluted in 500 μl of DMEM, which was then mixed with 500 μl of mouse blood and preheated at 37°C for 30 minutes. The mosquitoes were offered this mixture through a thin parafilm membrane using a circulating water system at 37°C for 30 minutes. After feeding, mosquitoes were chilled at 4°C for 10 minutes, and engorged mosquitoes were subsequently collected and maintained using a sugar solution.

Recombinant VSV virus and dsRNA were injected into ice-anesthetized female mosquitoes within 24 hours post-emergence using a Nanoliter 2000 injector (World Precision Instruments). Then, the mosquitoes were maintained on a sugar solution and cultured at 28°C with 70% humidity. For Zika virus (ZIKV MR766 strain) infection assay, the mosquitoes were blood-fed with ZIKV after microinjection with antagomirs for 3 days or with rVSVs for 6 days. The antagomirs of miR-10 were synthesized by GUANGZHOU RIBOBIO CO., LTD. (China). Newly emerged fruit flies (within 24 hours post-eclosion) were microinjected with 50 nl of rVSV (5 × 10^4^ FFU). Approximately three-day-old fall armyworm larvae were microinjected with 150 nl of rVSV (1.5 × 10^5^ FFU), while beetle larvae were injected with 500 μl of rVSV (5 × 10^7^ FFU). Samples were collected on the 7th day post-infection for TCID_50_ analysis.

One-day-old *Ae. aegypti* larvae were immersed in either rVSV-E93 (rVSV-E93-RB: rVSV-E93-RC = 1:1, 1 × 10^9^ FFU/mL) or 2% FBS DMEM for 48 hours, after which they were transferred to clean water. Larvae exhibiting GFP green fluorescence throughout their bodies were selected under a fluorescence microscope. Images were captured using an Olympus SZX16 microscope. Daily observations were made to record the pupation rate.

#### RNA extraction and RT-qPCR

Vero cells and samples of mosquitoes including whole body, head, legs, midguts, ovaries, thorax, fat body, Malpighian tubules, and wings were collected to extract the total RNA using Trizol reagent (Cat. 15596018, Beijing Genesand Biotech, China). RT-qPCR experiments were conducted following the instructions of the One-Step TB Green PrimerScript RT-PCR Kit (Cat. RR066A, Takara, Japan). The expression levels of miR-10 were quantified using the TB Green Premix Ex Taq II (Tli RNaseH Plus) (Cat. RR820A, Takara, Japan), following the reverse transcription protocol provided by Mir-X miRNA First-Strand Synthesis Kit (Cat. 638313, Takara, Japan). Primers used for RT-qPCR are listed in Table S1.

#### RNAi experiments

The double strand RNA (dsRNA) targeting *FoxO* gene was synthesized following the instructions of the T7 RiboMAX Express RNAi system (Promega). The coding region of the *GFP* gene was used to prepare the control dsRNA. Then, approximately 1.5 μg of the corresponding dsRNA were injected into the ice-anesthetized female mosquitoes that emerged within 24 h using a Nanoliter 2000 Injector (World Precision Instruments). Three days post-injection, 5-10 injected mosquitoes were collected to detect FoxO expression at both the mRNA and protein levels, and the remaining mosquitoes were used for the subsequent assays.

#### Confocal microscopy

Virus-infected cells or mosquito midguts were fixed in 4% paraformaldehyde for one hour at room temperature. Permeabilization was achieved using PBS with 0.5% Triton X-100 at room temperature for 10 minutes. Subsequently, samples were stained with Hoechst 33342 (Cat. C0031, Solarbio Life Sciences, China) to visualize the nuclei.

For the fluorescence detection of rVSV infection in mosquito tissues, midgut, brain, leg, and fat body samples were dissected at 7 dpi. Following PBS washing, these tissues were fixed in 4% paraformaldehyde for one hour at room temperature. Images were then visualized under a LEICA Stellaris 5 confocal microscope.

### Quantification and statistical analysis

All statistical analyses were conducted using GraphPad Prism 8 software (GraphPad Software, USA). Data are presented as mean ± SD unless otherwise specified. Statistical test methods included the Kruskal-Wallis test followed by Dunn's post hoc tests, two-sided unpaired *t*-test, one-way ANOVA test followed by Dunnett's post hoc tests, Chi-square test followed by Bonferroni test, Kaplan-Meier curves and log-rank test as shown in each figure legend. To ensure the reproducibility of our findings, each experiment was independently replicated at least three times. Sample sizes for each experiment were determined based on preliminary data and power analysis to ensure adequate statistical power to detect significant differences. All of the statistical details of experiments can be found in the figure legends. No data points were excluded from the analyses. Controls were consistently included in all experiments to validate the results. Insects were randomly assigned to experimental groups. No blinding was implemented in this study. We solely focused on measurable variables such as hatch rate, number of eggs, and survival rate.
