# Supplementary material for: Gene delivery in mosquitos with a vesicular stomatitis virus vector
Source: iScience. 2025 Sep 7;28(10):113510. doi: 10.1016/j.isci.2025.113510 (PMC12546990; doi:10.1016/j.isci.2025.113510)
Supplement: Document S1. Figures S1–S3 and Table S1 [file mmc1.pdf]

iScience, Volume *28*

Supplemental information

Gene delivery in mosquitos

with a vesicular stomatitis virus vector

Yuhang Zhang, Xueli Wang, Huiying Qi, Fei Yuan, Hongyue Li, Qiang Hu, Zhen Zou, and Aihua Zheng

Supplemental information

Supplementary figures and figure legends

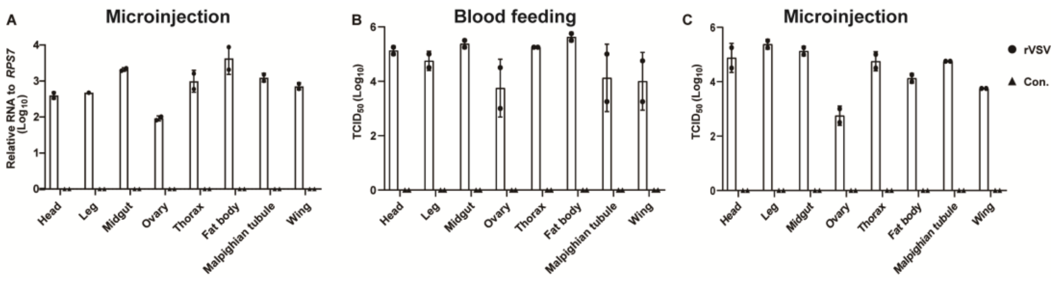

Figure S1. Tissue preference of rVSV infection in *Ae. aegypti*, related to Figure 1. (A) Female mosquitoes within 24 hours post-emergence were microinjected with 1 × 10^3 FFU rVSV. Total RNAs from eight tissues of fifteen mosquitoes were extracted at 7 dpi, and the viral RNAs were detected by RT-qPCR. The viral titers in different tissues of mosquitoes infected with rVSV via blood feeding (B) or microinjection (C) were measured using the $TCID_{50}$ assay at 7 dpi. Control (Con.) represents uninfected mosquito groups. These results are representative of two independent experiments.

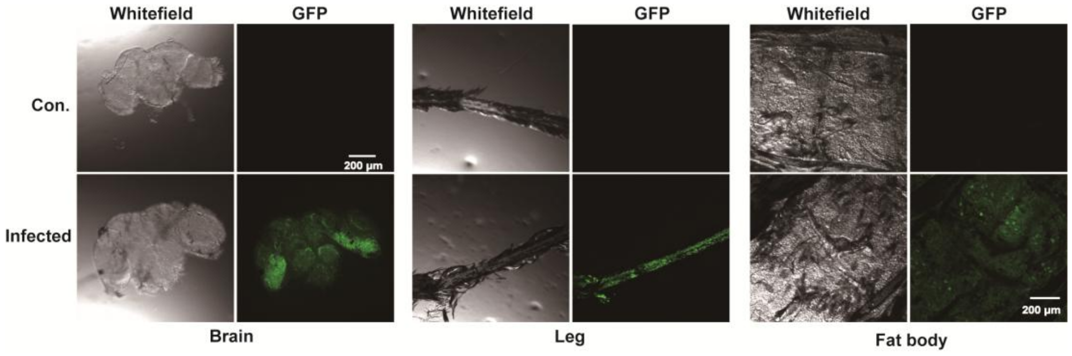

Figure S2. Fluorescence detection for mosquito tissues, related to Figure 1. At 7 dpi, mosquitoes that had been blood-fed with rVSV were dissected, then their brain, leg and fat body tissues were visualized under a LEICA confocal microscope. Control (Con.) represents uninfected mosquito groups. GFP was shown in green.

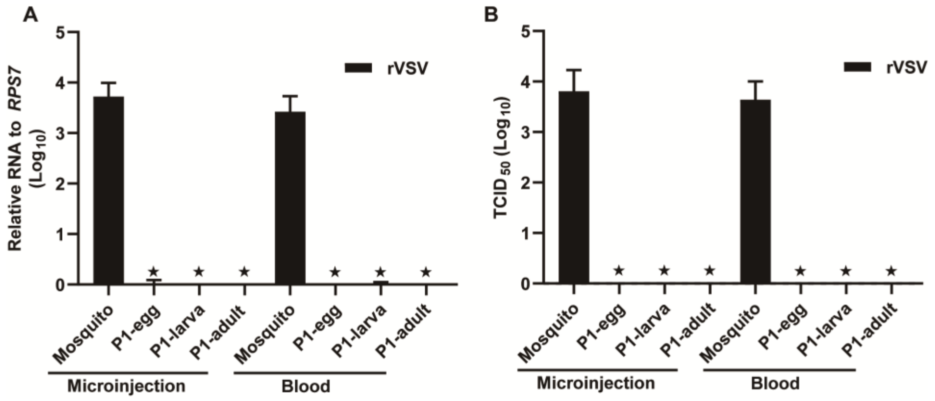

Figure S3. Vertical transmission of rVSV in mosquitoes, related to Figure 1. Twelve mosquitoes were microinjected or blood-fed with rVSV, and virus RNA levels (A) and infectious virion (B) of each mosquito and progeny were measured using RT-qPCR and $TCID_{50}$. Data are presented as means ± SD. Black stars (★) indicate virus titers under the detection limit. P1 indicates the offsprings of parental mosquitoes.

Table S1. Primers used in this study.

RT-qPCR	
Primer	Sequence (5' to 3')
RPS7-qRT-F	TCAGTGTACAAGAAGCTGACCGGA
RPS7-qRT-R	TTCCGCGCGCTCACTTATTAGATT
VSV-qRT-N-F	CGGAGGATTGACGACTAATGC
VSV-qRT-N-R	ACCATCCTAGCCATTCGAC
FoxO-qRT-F	CATCTGGAACCGGAATGTCT
FoxO-qRT-R	AACGGTACTGGGCGTAAGTG
E93-qRT-F	ACTGGAGCGCGTTGCTGAAGA
E93-qRT-R	AGGTTCAACGGTTGGCGCGT
GFP-qRT-F	AAGCAGAAGAACGGCATCAAGGTG
GFP-qRT-R	GGCGGTCACGAACTCCAGCA
β actin-qRT-F	CTCTCTTCCAACCTTCCTTCC
β actin-qRT-R	CAGACTCGTCATACTCCTGCTT
**RNAi**	
Primer	Sequence (5' to 3')
FoxO-T7-F	TAATACGACTCACTATAGGGGCTTGGGGTAACCTGTCGTA
FoxO-T7-R	TAATACGACTCACTATAGGGTGCTAGATCCGGTGGATAGG
FoxO-F	GCTTGGGGTAACCTGTCGTA
FoxO-R	TGCTAGATCCGGTGGATAGG
